# Strong Stackelberg reasoning in symmetric games: An experimental replication and extension

**DOI:** 10.7717/peerj.263

**Published:** 2014-02-25

**Authors:** Briony D. Pulford, Andrew M. Colman, Catherine L. Lawrence

**Affiliations:** 1 School of Psychology, University of Leicester, Leicester, UK; 2 School of Psychology, Bangor University, Bangor, UK

**Keywords:** Experimental games, Decision making, Coordination, Heuristics, Cooperation, Stackelberg reasoning

## Abstract

In common interest games in which players are motivated to coordinate their strategies to achieve a jointly optimal outcome, orthodox game theory provides no general reason or justification for choosing the required strategies. In the simplest cases, where the optimal strategies are intuitively obvious, human decision makers generally coordinate without difficulty, but how they achieve this is poorly understood. Most theories seeking to explain strategic coordination have limited applicability, or require changes to the game specification, or introduce implausible assumptions or radical departures from fundamental game-theoretic assumptions. The theory of strong Stackelberg reasoning, according to which players choose strategies that would maximize their own payoffs if their co-players could invariably anticipate any strategy and respond with a best reply to it, avoids these problems and explains strategic coordination in all dyadic common interest games. Previous experimental evidence has provided evidence for strong Stackelberg reasoning in asymmetric games. Here we report evidence from two experiments consistent with players being influenced by strong Stackelberg reasoning in a wide variety of symmetric 3 × 3 games but tending to revert to other choice criteria when strong Stackelberg reasoning generates small payoffs.

## Introduction

A well-known shortcoming of orthodox game theory is its inability to explain or justify strategic coordination between individuals in situations in which their interests coincide, so that they are motivated solely to coordinate their actions. Strategic coordination is a familiar phenomenon in a wide range of social activities, from teeter-tottering (see-sawing) and ballroom dancing to firefighting and launching nuclear missiles under the security requirements of the “two-man rule,” but game theory, in its standard form, provides no reason or justification for choosing the strategies that appear intuitively obvious to achieve successful coordination in many coordination games (Anderlini, 1999; Aumann & Sorin, 1989; Bacharach, 2006; Bardsley *et al.*, 2010; Colman, Pulford & Lawrence, *in* *press*; Cooper *et al.*, 1990; Crawford & Haller, 1990; Harsanyi & Selten, 1988; Janssen, 2001).

**Figure 1:**
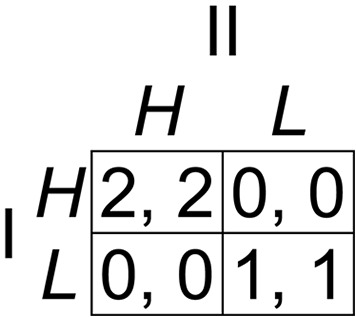
The Hi-Lo game. This game has a payoff-dominant Nash equilibrium at (*H*, *H*) and a payoff-dominated equilibrium at (*L*, *L*).

Consider a typical example of coordination between two software companies developing applications likely to be used in conjunction with each other. Each company has to decide independently of the other which of two standards to use for its application: HTML (*H*) or Linux Standard Base (*L*). It is in each company’s interest to use the same standard as the other so that the applications are compatible. Suppose that, on some measure of functionality or cost, *H* is twice as good as *L* for both applications. This scenario can be represented by the Hi-Lo payoff matrix depicted in Fig. 1. According to standard notation, Player I chooses a strategy represented by a row, either *H* or *L*, Player II independently chooses a strategy represented by a column, either *H* or *L*, and the outcome of the game is one of the four cells where the chosen strategies intersect, the first number in each cell representing the payoff to Player I and the second the payoff to Player II. Both players are motivated to coordinate their strategy choices, and the outcome (*H*, *H*), with payoffs of 2 to each player, is better for both than (*L*, *L*), with payoffs of 1 each. There is no element of conflict in this *pure coordination game*, because the players have identical preferences in every outcome.

The Hi-Lo game is the simplest example of a *common interest game*, in which one strategy profile or outcome strongly *payoff*-*dominates* all other possible outcomes, yielding better payoffs to each player than any other (Anderlini, 1999; Aumann & Sorin, 1989). This particular common interest game is a pure coordination game because the players’ preferences coincide exactly in every possible outcome. In pure coordination games such as this, optimal choices seem obvious, but they are not explained or justified by orthodox game theory. It seems intuitively rational for both players to choose *H* in the Hi-Lo game, resulting in the payoff-dominant outcome (*H*, *H*), but (remarkably) this conclusion cannot be derived from the standard common knowledge and rationality assumptions of game theory. These assumptions are (a) that the players know the specification of the game—in this case, the payoff matrix shown in Fig. 1—and everything that can be logically deduced from it; (b) that the players are instrumentally rational in the sense of always seeking to maximize their own payoffs; and (c) that this is all *common knowledge*, in the sense that both players know it, know that both know it, know that both know that both know it, and so on. From these assumptions we can infer that Player I has a reason to choose *H* if and only if there is a reason to expect Player II to choose *H*; but Player I has no reason to expect Player II to choose *H*, because the game is symmetric and Player II has a reason to choose *H* if and only if there is a reason to expect Player I to choose it. Any attempt to derive a reason for choosing *H* from the standard assumptions of game theory leads to an infinite regress. Nevertheless, experimental evidence has corroborated common sense by showing that, in practice, more than 96% of players successfully coordinate on the obvious payoff-dominant (*H*, *H*) outcome in the Hi-Lo game (Bardsley *et al.*, 2010). Research stretches back to 1960, when Schelling (1960) showed that human decision makers have a remarkable facility for coordinating their strategies in pure coordination and other common interest games, although coordination failures sometimes occur, especially in more complicated cases (Cooper *et al.*, 1990; Van Huyck, Battalio & Beil, 1990).

How can coordination be explained? In a two-player game, a *Nash equilibrium* is an outcome in which the strategy chosen by each player is a *best reply* to the strategy chosen by the other, a best reply being a strategy yielding the highest payoff to the player choosing it, given the strategy chosen by the co-player (Nash, 1950, 1951). Both (*H*, *H*) and (*L*, *L*) are Nash equilibria in the Hi-Lo game shown in Fig. 1, because *H* is obviously the best reply to *H*, and *L* is the best reply to *L*. To explain the intuitive appeal of (*H*, *H*), Harsanyi & Selten (1988) introduced a *payoff-dominance principle* as an axiom of rationality into their theory of equilibrium selection in games. According to this principle, it is simply an axiomatic feature of human rationality that players will choose a payoff-dominant equilibrium if it exists.

**Figure 2:**
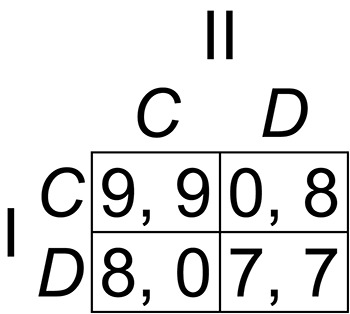
The Stag Hunt game. This game has a payoff-dominant Nash equilibrium at (*C*, *C*) and a payoff-dominated Nash equilibrium at (*D*, *D*).

Several years later, after a further consideration of the Stag Hunt game shown in Fig. 2, Harsanyi (1995) abandoned the payoff-dominance principle altogether, acknowledging that it does not provide a reason for choice and is therefore not a useful element of the theory of equilibrium selection. Some common interest games have only one Nash equilibrium payoff-dominating all other (non-equilibrium) outcomes of the game, and the Harsanyi–Selten payoff-dominance principle is, in any case, powerless to explain the coordination and payoff dominance phenomena in these cases, because it applies only when one equilibrium dominates another equilibrium or other equilibria. All theories of coordination predict that players will select a payoff-dominant Nash equilibrium whenever one exists in a game, and the payoff-dominance principle was therefore only a description, and not an explanation, of the phenomenon. What needs to be explained is *why* and *how* players choose payoff-dominant Nash equilibria.

It has sometimes been argued that *salience* alone is enough to enable players to coordinate. In a common interest game, by definition, one outcome is better for both players than every other, and that outcome is therefore salient in the sense that sticks out from the others in a way that each player notices and expects the other player to notice. It may seem that its salience provides a sufficient clue to enable the players to coordinate their choices. For example, in the Hi-Lo game (Fig. 1), each player is likely to notice that the (*H*, *H*) outcome is salient by virtue of yielding both players their highest payoffs, and this may seem to be all that is required to lead each player to choose the *H* strategy. But Player I’s choice of *H* does not, in itself, bring about the outcome that pays both players their highest payoffs, because that requires Player II to choose *H* also, and if Player II were to choose *L*, then *L* would have be a better choice for Player I. To work out what it is rational to do, Player I must form a belief about what Player II is likely to do. Player I cannot be confident that choosing *H* will bring about the outcome in which both players choose *H* unless there is a reason to expect Player II to choose *H*. But the proposition that salience alone provides such a reason is precisely what the argument from salience seeks to prove. In a frequently cited article, Gilbert (1989) analyzed the logic of the salience argument in detail and concluded that “mere salience is *not* enough to provide rational agents with a reason for action” (p. 69), and Sugden (1993) has provided a further detailed refutation of the notion that salience alone suffices to enable coordination in common interest games.

A number of explanations have been suggested. Some of these rely on altering the rules of the game that specify single, independent strategy choices by allowing repetitions (Aumann & Sorin, 1989; Anderlini & Sabourian, 1995) or costless pre-play “cheap talk” between players (Anderlini, 1999; Ellingsen & Östling, 2010; Farrell, 1988; Rabin, 1994). Social projection theory (Acevedo & Krueger, 2005; Krueger, 2007; Krueger & Acevedo, 2005; Krueger, DiDonato & Freestone, 2012) assumes that players use a form of evidential decision theory according to which people expect their co-players to choose whatever they themselves choose, but evidential decision theory was excoriated by Lewis (1981) and is still generally viewed with skepticism (Chater & Vlaev, 2012; Yamagishi, 2012).

The most influential theories require assumptions that are only slightly less radical. According to cognitive hierarchy theory (Camerer, Ho & Chong, 2004), players reason with varying levels of strategic depth, Level-0 players choosing strategies randomly, Level-1 players maximizing their own payoffs given their belief that their co-players are Level-0 players, Level-2 players maximizing their own payoffs given their belief that their co-players are Level-1 or Level-0 players, and so on. This theory explains coordination in the Hi-Lo game by Level-1 players who expect their co-players to choose randomly and who therefore do better for themselves, on average, by playing *H* than *L*, and Level-2 players who expect their Level-1 co-players to choose *H*, for the reason just explained, and who therefore do better for themselves by also choosing *H*, and so on, but it requires the implausible assumption that all apart from Level-0 players invariably assume that their co-players reason with less strategic depth than themselves. A more serious problem with this theory is that it fails to explain coordination in some important common interest games. A famous example, discussed at length by Harsanyi & Selten (1988) is the version of the Stag Hunt game shown in Fig. 2, in which a Nash equilibrium at (*C*, *C*) payoff-dominates a second equilibrium at (*D*, *D*). A Level-1 player acts as though believing that the Level-0 co-player will choose randomly and therefore chooses *D*, because (1/2 × 8) + (1/2 × 7) > (1/2 × 9) + (1/2 × 0); a Level-2 player, acting as though believing that the Level-1 co-player will choose *D* (for the reason just given) also chooses *D*, because 7 > 0; and the same applies to higher levels: players fail to coordinate on the payoff-dominant outcome at any level of strategic reasoning.

According to theories of team reasoning (Bacharach, 1999; Bacharach, 2006; Sugden, 1993; Sugden, 2005) there are circumstances in which players are motivated to maximize the *collective* payoff of the players involved in the game, rather than their individual payoffs. In the Hi-Lo game shown in Fig. 1, assuming naturally that the collective payoff is simply the sum of the individual payoffs, the team-reasoning solution is (*H*, *H*) because the collective payoff of 4 is greater there than in any other outcome, and in the Stag Hunt game shown in Fig. 2, it is (*C*, *C*) for the same reason. There is experimental evidence for team reasoning (Bardsley *et al.*, 2010; Colman, Pulford & Rose, 2008), and more general evidence for cognitive gains from collective rationality in decision making (Curşeu, Jansen & Chappin, 2013). The main problem with theories of team reasoning is that players are assumed to replace the (individual) payoffs in the game, shown in the payoff matrix, with collective payoffs. This requires the abandonment of methodological individualism, a cornerstone of decision theory and game theory, according to which decision makers and players choose the options that are best for themselves, given their individual preferences (Elster, 1982).

### Strong Stackelberg reasoning

The theory of strong Stackelberg reasoning (Colman, Pulford & Lawrence, *in* *press*), an improved version of an earlier theory (Colman & Bacharach, 1997), provides an explanation of coordination in all dyadic (two-player) common interest games, and in particular, it provides an explanation of why players tend to choose strategies associated with a payoff-dominant Nash equilibrium. It requires no modification of the rules of the game (no repetitions or cheap talk), it adheres to the standard knowledge and rationality assumptions of game theory, and it incorporates the assumption of methodological individualism; thus it avoids the problems associated with competing theories. Its distinctive assumption is that players behave as though their co-players will anticipate any strategy choice and invariably choose a best reply to it. In other words, players behave as though they were choosing first in a sequential-choice game with *perfect information*—a game in which the co-player, moving second, knows their previous move. Players choose strategies that maximize their own payoffs, given that assumption, whenever their own and their co-players’ strategies form Nash equilibria. When the Stackelberg strategies resulting from this form of reasoning do not form Nash equilibria, the theory makes no predictions, because a non-equilibrium outcome is inherently unstable, leaving at least one player with a reason to choose differently and thereby achieve a better payoff. Strong Stackelberg reasoning is a simple theory, according to which players in dyadic games choose strategies that would maximize their own payoffs if their co-players could invariably anticipate their strategy choices and play counter-strategies that yield the maximum payoffs for themselves. The key assumption is relatively innocuous, first because game theory imposes no constraints on players’ beliefs, apart from consistency requirements, and second because the theory does not assume that players necessarily believe that their strategies will be anticipated, merely that they behave as though that were the case, as a heuristic aid to choosing the best strategy. Strong Stackelberg reasoning is, in fact, merely a generalization of the *minorant* and *majorant* models introduced by Von Neumann & Morgenstern (1944); section 14.4.1, pp. 100–104) and used to rationalize their solution of strictly competitive games.

The improved version of the theory assumes that best replies involved in Stackelberg reasoning are strong in the sense of Harsanyi & Selten (1988), so that there are never two or more equally good best replies, a condition that is necessarily satisfied provided that players are not totally indifferent to the payoffs of their co-players (Colman, Pulford & Lawrence, *in* *press*). The earlier version of the theory (Colman & Bacharach, 1997), lacking this assumption, breaks down and fails to generate unique Stackelberg strategies when two or more best replies yield the same payoff to one player but different payoffs to the other. If best replies are strong, then unique Stackelberg strategies are generated, and if they are in Nash equilibrium, then the game is *S-soluble*. If best replies are not strong, or if Stackelberg strategies are generated but are not in Nash equilibrium, then the game is *non-S-soluble*. Strong Stackelberg reasoning thus involves a Stackelberg strategy generator (choosing the best strategy given an assumption that the co-player will anticipate any choice and play a best reply to it) followed by a Nash filter (checking that the resulting Stackelberg strategies generated for the players are in equilibrium). If the game is S-soluble, then Stackelberg-reasoning players choose and play their Stackelberg strategies, and the resulting outcome is the *Stackelberg solution*. If the game is non-S-soluble, then the theory makes no specific predictions. A formalization and mathematical development of the theory have been provided elsewhere (Colman, Pulford & Lawrence, *in* *press*; Colman & Bacharach, 1997).

Applying strong Stackelberg reasoning to the Hi-Lo game shown in Fig. 1, Player I behaves as though any strategy choice will be anticipated by Player II, so that Player I’s *H* would be met by the unique best reply *H*, and *L* would be met by the unique best reply *L*. Player I receives a payoff of 2 in the first case and 1 in the second, therefore Player I’s payoff-maximizing Stackelberg strategy is *H*. Because the game is symmetric, the same applies to Player II. The strategy pair (*H*, *H*) generated by this reasoning process is a Nash equilibrium, therefore both players choose *H*. In the Stag Hunt game shown in Fig. 2, both players choose *C* following a similar process of strong Stackelberg reasoning. It has been proved (Colman & Bacharach, 1997) that every common interest game is S-soluble, and that if a game with multiple Nash equilibria has one equilibrium that payoff-dominates the others, then its Stackelberg solution is the payoff-dominant equilibrium. Strong Stackelberg reasoning therefore provides an explanation for coordination in all dyadic common interest games.

The theory of virtual observability (Weber, Camerer & Knez, 2004) incorporates some ideas reminiscent of strong Stackelberg reasoning. Virtual observability occurs in games in which Player I chooses a strategy before Player II, but Player II chooses in ignorance of Player I’s earlier choice. The theory was designed to explain timing effects in games with asymmetric equilibria, Player I preferring one equilibrium and Player II another, and experimental evidence confirmed a small first-mover advantage. The theory of strong Stackelberg reasoning applies to simultaneous-choice games without timing manipulation, and it is relevant to games with symmetric equilibria as well as those with asymmetric equilibria. However, the two theories are related, because strong Stackelberg reasoning involves acting *as though* strategy choices were sequential.

Strong Stackelberg reasoning seems a natural and intuitive form of reasoning in common interest games. It is an example of a *simulation heuristic*, a class of heuristics first identified by Kahneman & Tversky (1982), whereby people solve problems by running mental simulations. How easily a mental model reaches a particular outcome helps a decision maker to judge how likely it is for that outcome to occur in the actual situation. Kahneman and Tversky provided experimental evidence that human decision makers use simulation heuristics to predict the behavior of others in certain circumstances, and to answer questions about what might have happened in different circumstances, by mentally “undoing” events that occurred and then running mental simulations with the relevant input parameters of the simulation model altered. According to the theory of strong Stackelberg reasoning, players solve coordination problems in common interest games by performing mental simulations of what would occur if (counterfactually) they had the first move and their co-players could move second, with knowledge of their preceding move. This approach seems natural when standard reasoning fails to provide an answer and salience, on its own, provides no reason for choice.

### Previous experimental evidence

An experimental investigation of strategy choices in simple common interest games such as the Hi-Lo game shown in Fig. 1 cannot provide a stringent test of the theory of strong Stackelberg reasoning, because in such games Stackelberg solutions tend to be intuitively obvious. More interesting and diagnostic are games in which strong Stackelberg reasoning makes clear predictions that are not obvious without help from the theory. Colman & Stirk (1998) tested the theory experimentally in all 12 ordinally non-equivalent symmetric 2 × 2 games, nine of which happen to be S-soluble and the other three non-S-soluble. Players tended to choose Stackelberg strategies in S-soluble games, with large effect sizes in every case, whereas choices in the non-S-soluble games were variable, some biased toward and others away from the Stackelberg strategies, with much smaller effect sizes. Colman, Pulford & Lawrence (*in* *press*) reported the results of two experiments using asymmetric 3 × 3 and 4 × 4 games, designed to test cognitive hierarchy, team reasoning, and strong Stackelberg reasoning theories against one another in games without obvious, payoff-dominant solutions. These experiments provided further evidence for Stackelberg reasoning, although both experiments suggested that cognitive hierarchy Level-1 reasoning and team reasoning were also frequently used by players.

In the experiment reported by Colman & Stirk (1998), six of the nine symmetric S-soluble games had Stackelberg strategies that were also strongly dominant. Strong *strategic dominance*, not to be confused with payoff dominance, exists when a strategy is an unconditionally best, sure-thing strategy that yields a higher payoff to the player choosing it than another strategy, irrespective of the co-player’s choice. Rational players never choose strongly dominated strategies, therefore players may have chosen Stackelberg strategies in these nine S-soluble games because they were strongly dominant and not necessarily because they were Stackelberg strategies. This confounding problem cannot be avoided in symmetric 2 × 2 games. Although the problem was eliminated by Colman, Pulford & Lawrence (*in* *press*), using asymmetric 3 × 3 and 4 × 4 games without dominant strategies, and evidence for strong Stackelberg reasoning was once again found, it is clear that the theory is supported by very shallow experimental foundations, and further evidence is required to secure its evidential base and establish its validity with confidence. In particular, bearing in mind the technical limitations of the symmetric 2 × 2 games that Colman and Stirk used, especially the fact that confounding with strong strategic dominance cannot be avoided, and the fact that Colman, Pulford, and Lawrence had to use asymmetric games because of the nature of their investigation (comparing different theories), it would be useful to establish whether strong Stackelberg reasoning occurs in other symmetric games. In addition, and in light of growing concerns about the risks of Type 1 errors and discussions of the need for replication studies (Francis, 2012; Koole & Lakens, 2012; Pashler, Coburn & Harris, 2012; Ritchie, Wiseman & French, 2012), it seems important to determine whether these findings can be replicated with completely different games.

### Rationale for further experiments

The explanation of coordination in common interest games is an important unanswered scientific problem because it exposes one of the most obvious shortcomings of orthodox game theory. Several competing theories, reviewed in the paragraphs above, have attempted to explain coordination and payoff dominance, and the theory of strong Stackelberg reasoning provides an explanation that avoids serious problems associated with the competing theories. It is impossible to test the theories against one another in common interest games, because all theories would obviously make the same prediction in every such game, namely that players will select strategies associated with the payoff-dominant Nash equilibrium. Furthermore, it is impossible to test the theories against one another in symmetric games, because it turns out to be impossible to construct symmetric games in which different theories predict distinct choices. In order to derive diverging predictions from competing theories, it turns out to be necessary to use asymmetric experimental games, and the results of research using asymmetric games in which the leading theories all predict different choices has suggested that strong Stackelberg reasoning may influence some players (Colman, Pulford & Lawrence, *in* *press*). It is nevertheless important to test the theory in symmetric games also, because the earlier research leaves open the question of whether this form of strategic reasoning occurs in symmetric games, especially bearing in mind that it is the phenomenon of coordination in symmetric games such as the Hi-Lo game (Fig. 1) that first generated interest and stimulated the development of theories of coordination in the first place.

A small first-mover advantage effect has been shown to occur in virtually observable sequential games with asymmetric equilibria (Weber, Camerer & Knez, 2004), but it is less obvious why any such effect should occur in non-sequential (simultaneous-move) games with only symmetric equilibria. The theory of strong Stackelberg reasoning is specifically intended to provide an explanation for selection of payoff-dominant equilibria in common interest games such as the Hi-Lo game (Fig. 1), and this game, like many other interesting and important common interest games, is a symmetric game with symmetric Nash equilibria. According to the theory of strong Stackelberg reasoning, players derive a reason for selecting payoff-dominant equilibria in all common interest games, even in games in which such equilibria are symmetric, by using a form of sequential reasoning. If the theory of strong Stackelberg reasoning is to provide a convincing explanation of coordination in common interest games, then it is necessary to show that players use this form of strategic reasoning in games with symmetric equilibria, as well as games with asymmetric equilibria, such as those investigated by Colman, Pulford & Lawrence (*in* *press*).

The experiments reported in this article are required because the only published experiment designed to determine whether decision makers use Stackelberg reasoning in symmetric games (Colman & Stirk, 1998) was restricted to symmetric 2 × 2 games, and in S-soluble 2 × 2 games Stackelberg strategies are almost invariably also strongly dominant strategies, so that the experiment provided virtually no independent evidence of strong Stackelberg reasoning that cannot be explained by dominant strategy selection. In order to check whether strong Stackelberg reasoning occurs in symmetric games, we have therefore constructed sets of symmetric 3 × 3 experimental games in which strong Stackelberg strategies are never dominant strategies, thus eliminating the confound in the earlier research.

## Experiment 1

The aim of this experiment was to test the theory of strong Stackelberg reasoning in a new set of symmetric 3 × 3 games, some S-soluble and some non-S-soluble, avoiding games with strongly dominant Stackelberg strategies. The principal hypothesis was that players would tend to choose Stackelberg strategies in the S-soluble games. A secondary hypothesis was that players would be less attracted to Stackelberg strategies in non-S-soluble games.

### Materials and Methods

The participants were 72 students and employees at the University of Leicester (50 female, 22 male), aged 18–47 years (*M* = 22.03, *SD* = 6.73), recruited from the School of Psychology’s participant panel. They were remunerated with the average of the payoffs in the 12 games that they played during the testing session. The payoffs in each of the 12 games ranged from zero to £5.00 ($8.00).

**Figure 3:**
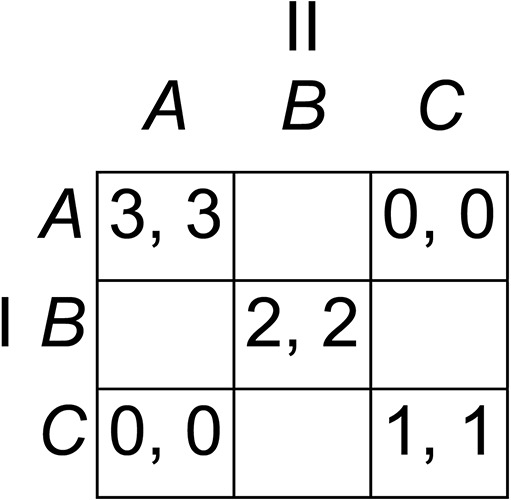
Game-generating template. This template was used for generating S-soluble and non-S-soluble symmetric 3 × 3 games for Experiment 1.

**Figure 4:**
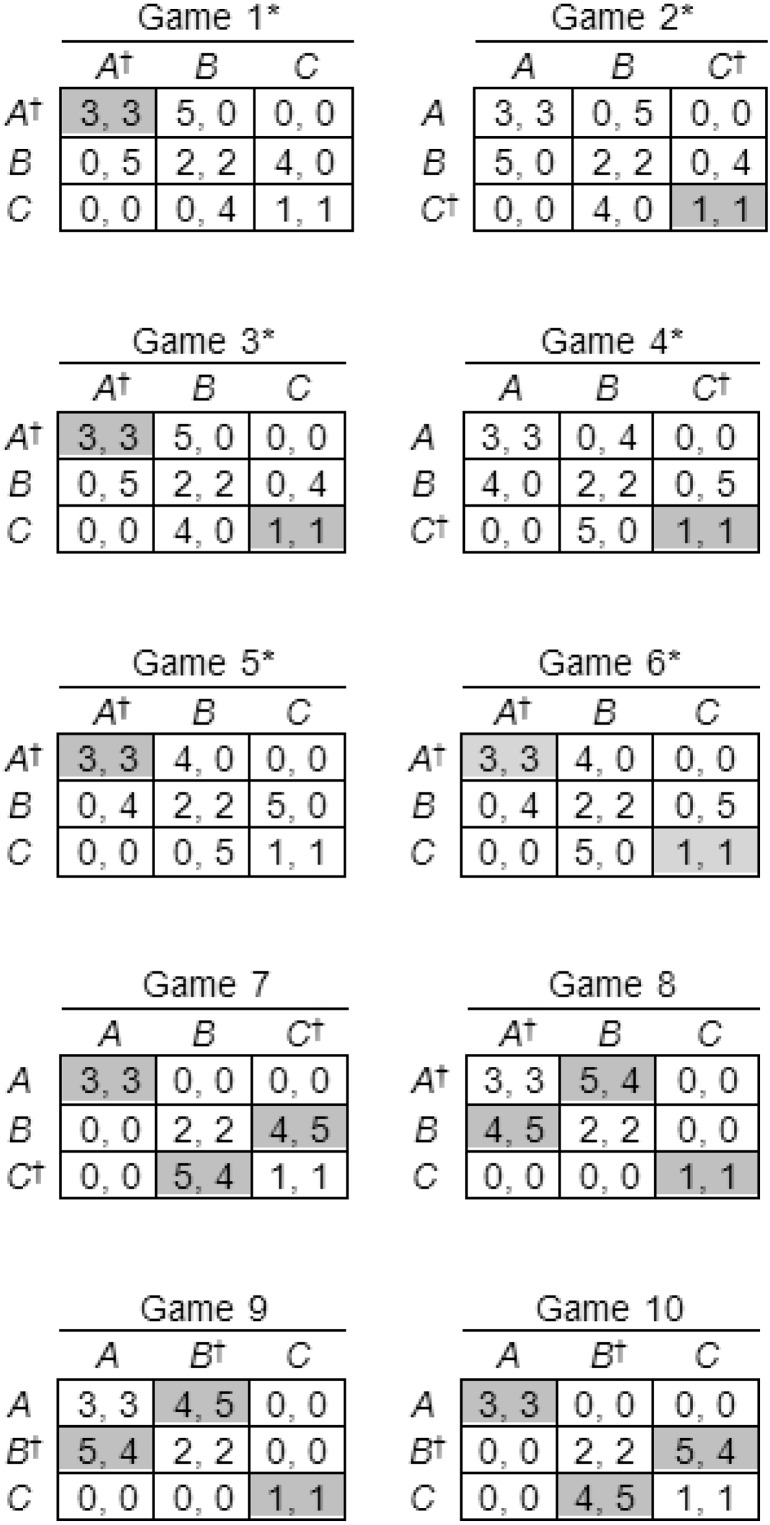
Games used in Experiment 1. Nash equilibria are shaded, S-soluble games indicated by asterisks, and Stackelberg strategies indicated by daggers. Player labels have been removed to save space.

We generated symmetric 3 × 3 games according to the following algorithm, designed to generate suitable games automatically, without the unintentional biases that might be introduced if games were constructed arbitrarily. Beginning with the template shown in Fig. 3, we inserted an arbitrary payoff to Player I of either 4 or 5 in one of the empty cells, then we inserted the same payoff to Player II in the symmetrically corresponding cell to maintain the symmetry of the game, followed by a payoff to Player II of 5 or 4 (different from the payoff to Player I) in a different empty cell, and a symmetrically corresponding payoff to Player I, and finally we filled the remaining spaces with zero payoffs. There are initially four empty cells in the template in Fig. 3, and therefore four spaces available for the payoff of 4 or 5 to Player I, and for each of these there remain three unfilled spaces for the payoff of 5 or 4 to Player II. Therefore, there are 12 permutations, the remaining payoffs being fully determined by the symmetry of the game after these two insertions. Of the 12 games generated by this algorithm, eight are S-soluble and four non-S-soluble. In two of the S-soluble games, the Stackelberg strategies *weakly* dominate both other strategies, and we therefore eliminated these two games. Weak dominance occurs when a strategy yields payoffs that are at least as high (including equality) against each of the co-player’s strategies, and strictly higher against at least one. Participants were presented with all 12 games, but we eliminated these two games from our data analysis. The remaining 10 games, displayed in Fig. 4, are the experimental games used in Experiment 1.

We conducted experimental sessions in even-numbered groups of up to 12, each player being paired with the same co-player throughout the testing session (fixed-matching protocol). Games were presented to players in printed booklets, with the labels and payoffs for Players I and II in different colors as an aid to understanding. In this experiment, strategy labels were not counterbalanced; the games were always presented as in Fig. 4. Each pair played the 12 experimental games (including the two excluded from data analysis) in a different random order. Written instructions are given in Appendix A. After all 12 games had been completed, participants recorded their demographic details. They were paid what they had earned at a prearranged meeting after the payoffs had been calculated.

### Results^3^ 3Raw data for Experiment 1 are available in the Supplementary Materials.


We performed a binary logistic regression analysis to predict choices of Stackelberg strategies (1 = Stackelberg strategy choice, 0 = non-Stackelberg strategy choice), using participant identity, participant age, participant sex, game number, value of the payoff if both players choose Stackelberg strategies, and number of equilibria in the game as predictor variables. After fitting a model we tested it against a constant model, and the difference was statistically significant: *χ*^2^(12) = 155.92, *p* < .001. The value of the Nagelkerke *R*^2^ = .27 indicates a medium-sized strength of relationship between model predictions and Stackelberg strategy choices.The coefficient of determination *R*^2^ summarizes the proportion of variance in the dependent variable associated with the predictor variables. In regression models with categorical dependent variables, it is not possible to compute a single statistic with all the characteristics of *R*^2^ in the linear regression model, but the Nagelkerke *R*^2^ provides a suitable approximation (Nagelkerke, 1991). Prediction success was 73% overall (57% for choice of non-Stackelberg strategies and 83% for choices of Stackelberg strategies). According to the Wald chi-square statistic, the only predictor variable that contributed significantly to the prediction was game number, *χ*^2^(9) = 120.23, *p* < .001, with some games eliciting more frequent Stackelberg strategy choices than others. Participant variables (identity, age, and sex), and other game variables (the value of the payoff if both players choose Stackelberg strategies and the number of equilibria in the game) did not have significant additional effects in the model independent of differences between the games.

The symmetric games used in this experiment, and Experiment 2 described below, preclude the possibility of comparing strong Stackelberg reasoning with other theories of coordination and payoff dominance, such as cognitive hierarchy (Level-*k*) theory and team reasoning, because different theories make different predictions in only a few asymmetric games. However, previous research (Colman, Pulford & Lawrence, *in* *press*), in which such model comparisons were made in asymmetric games, has already suggested that strong Stackelberg reasoning may be used, independently of other reasoning processes, by a substantial proportion of players.

The percentages of Stackelberg strategies chosen in S-soluble and non-S-soluble games are shown in Table 1, together with chi-square goodness-of-fit tests against a null hypothesis of random choice (one-third Stackelberg strategy choices and two-thirds other strategies), and effect sizes estimated using Cohen’s index *w* (Cohen, 1992), and choice percentages across all three strategies are shown in Table 2. The results are clearly consistent with our principal hypothesis: in five of the six S-soluble games, strategy choices were biased toward the options associated with Stackelberg strategies. This bias was large and significant in four of the five games (Games 1, 3, 5, 6) and small and non-significant in Game 4. Only in Game 2 was there a significant bias away from the Stackelberg strategy, and the effect size was small. The mean effect size in S-soluble games, excluding the anomalous Game 2 (because it would be misleading to count an effect in the wrong direction), was *w* = 0.86 (large). It is not difficult to explain why Games 2 and 4 produced results that differed from the others. They are the only two S-soluble games in which the Stackelberg solution yields very small payoffs of (1, 1) to the players, compared to (3, 3) in all other S-soluble games. Furthermore, in all other S-soluble games, the sum of payoffs to both players was greatest in the outcome corresponding to the Stackelberg solution. This suggests that collective rationality or team reasoning (Bacharach, 1999; Bacharach, 2006; Bardsley *et al.*, 2010; Sugden, 1993; Sugden, 2005) may have played a part in influencing the players’ choices. Symmetric games are not suitable for distinguishing between team reasoning and strong Stackelberg reasoning; however, previous research with asymmetric games (Colman, Pulford & Lawrence, *in* *press*) has suggested that players use both methods of reasoning, strong Stackelberg reasoning more frequently in simple games and team reasoning more frequently in complicated games.

**Table 1:** Choice of strategies in S-soluble and non-S-soluble games: Experiment 1. Choice of Stackelberg (S) and non-Stackelberg (N) strategies in S-soluble and non-S-soluble games: Experiment 1 (N = 72).

Game	% S	*χ*^2^(1)	*p*<	*w*	Bias toward
S-soluble					
1	88.89	99.18	.005	1.17	S
2	20.83	5.27	.025	0.27	N
3	86.11	89.47	.005	1.11	S
4	39.89	0.93	*ns*	0.11	S
5	77.78	63.35	.005	0.94	S
6	79.17	67.39	.005	0.97	S
Non-S-soluble					
7	47.22	6.06	.025	0.29	S
8	76.39	59.43	.005	0.91	S
9	51.39	10.31	.005	0.38	S
10	62.50	27.14	.005	0.61	S

**Notes.**

% S = percentage of players choosing Stackelberg strategies. Cohen’s (1992) effect size index *w* ≥ 0.10 is small, *w* ≥ 0.30 is medium, *w* ≥ 0.50 is large.

**Table 2:** Choice percentages across all three strategies in S-soluble and non-S-soluble games: Experiment 1.

Strategy choice	1^*^	2^*^	3^*^	4^*^	5^*^	6^*^	7	8	9	10
*A*	88.89^a^	37.50	86.11^a^	29.17	77.78^a^	79.17^a^	4.17	76.39^a^	47.22	15.28
*B*	11.11	41.67	11.11	31.94	20.83	2.78	48.61	23.61	51.39^a^	62.50^a^
*C*	0.00	20.83^a^	2.78	38.89^a^	1.39	18.06	47.22^a^	0.00	1.39	22.22

* S-soluble games

a Stackelberg strategies

The results are also broadly consistent with our secondary hypothesis, that players would be less attracted to Stackelberg strategies in non-S-soluble games, because in these games strong Stackelberg strategies intersect in outcomes that are not Nash equilibria and, according to the definition of a Nash equilibrium, such outcomes are inherently unstable, providing at least one player with an incentive to choose differently to ensure a better payoff. In the four non-S-soluble games, although choices were biased toward the options associated with Stackelberg strategies, the effect sizes are smaller (mean *w* = 0.55). Players chose these strategies more frequently in S-soluble games (*M* = 65.44%) than in non-S-soluble games (*M* = 59.38%), although this difference is non-significant. Players were attracted by the option associated with the Stackelberg strategy in the non-S-soluble Game 7 in spite of the small payoff of (1, 1) in the non-equilibrium outcome where the Stackelberg strategies intersect. However, in that game they were probably influenced by the prospect of the highest payoff in the (5, 4) outcome, and it may have seemed feasible, because the co-player would receive the second-highest possible payoff in that outcome.

### Reasons for Experiment 2

The fact that the bias toward Stackelberg strategies was larger in the S-soluble games than in the non-S-soluble games tends to suggest that strong Stackelberg reasoning influenced players more in the S-soluble games, as predicted by the theory. However, results for two of the six S-soluble games failed to corroborate the principal hypothesis, and our method of generating experimental games produced only four non-S-soluble games, therefore it would be useful to check the findings with a larger and more varied selection of symmetric S-soluble and especially non-S-soluble games.

Another drawback of our game-generating procedure was that it did not exclude weakly dominant strategies altogether. We eliminated two games in which Stackelberg strategies weakly dominated both other strategies, creating a potential confound with strong Stackelberg reasoning. But in two of the six remaining S-soluble games (Games 3 and 6), the Stackelberg strategy (*A*) weakly dominates *one* of the other strategies (*B*). A further replication, using games without any (even weakly) dominant strategies at all is therefore desirable.

**Figure 5:**
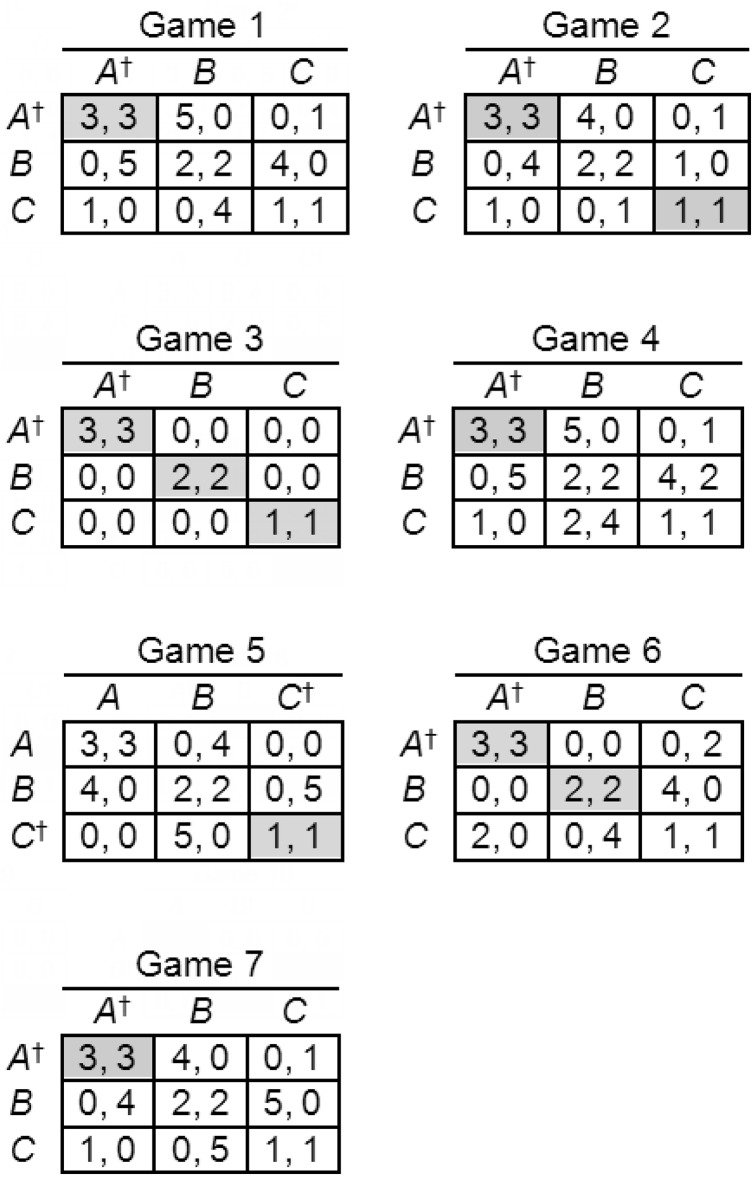
S-soluble games used in Experiment 2. Nash equilibria are shaded and Stackelberg strategies indicated by daggers. Player labels have been removed to save space.

**Figure 6:**
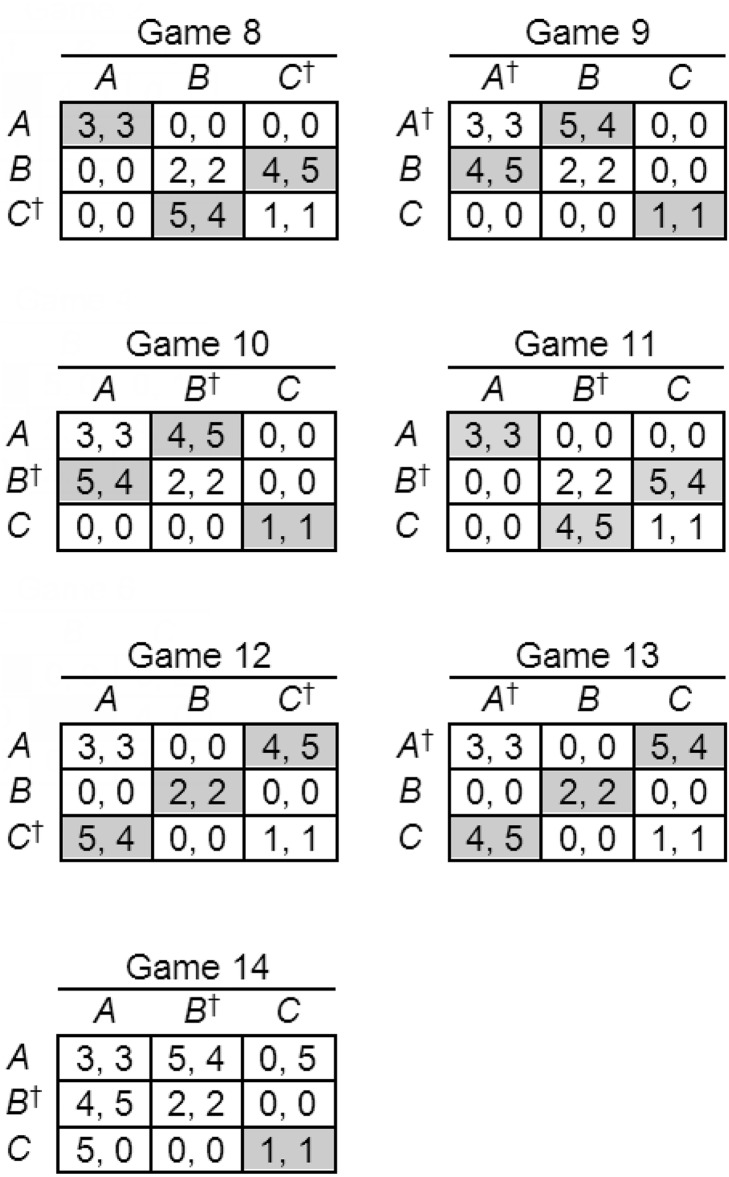
Non-S-soluble games used in Experiment 2. Nash equilibria are shaded and Stackelberg strategies indicated by daggers. Player labels have been removed to save space.

In game theory, the labeling and positioning of rows and columns of a payoff matrix have no effect on a game’s strategic properties, but permutations may influence the responses of human decision makers, especially in symmetric games, because symmetry is most obvious when a matrix is presented in what we call *root position*, as in the versions shown in Figs. 4–6. It would therefore be useful to replicate Experiment 1 with the additional control of randomized permutation of rows and columns.

Experiment 1 used a fixed-matching protocol: each player was matched with the same co-player for all games. In order to avoid carry-over effects between games, it is sometimes considered preferable to match each player anonymously with a different co-player for each game (random-matching protocol). It is also desirable to motivate the players with slightly larger monetary incentives than in Experiment 1, because there is evidence that more generous monetary incentives reduce the variance in behavior and generally improve decision making, often bringing decisions closer to game-theoretical predictions (Camerer & Hogarth, 1999; Smith & Walker, 1993). Furthermore, monetary incentives produce the greatest performance improvements in decision tasks of intermediate difficulty (Hertwig & Ortmann, 2001), and 3 × 3 games seem to fall precisely into that category. In Experiment 2, we used larger incentives and also incorporated the other improvements mentioned in the preceding paragraphs.

## Experiment 2

Experiment 2 was designed to test the theory of strong Stackelberg reasoning in a fresh set of symmetric 3 × 3 games without any weak or strong dominant strategies at all. In addition, we implemented an anonymous random-matching protocol, we randomized the rows and columns of the payoff matrices, and we introduced larger incentive payments. To throw more light on the players’ reasoning, we collected verbal accounts of their reasons for their choices. The set of games used in Experiment 2 includes equal numbers of S-soluble and non-S-soluble games, to provide a broader basis of comparison. In this computer-controlled experiment, we did not provide participants with feedback regarding their co-players’ choices until the end of each testing session.

### Materials and Methods

The participants were 127 students and employees at the University of Leicester (32 male, 95 female), aged 18-53 years (*M* = 22.78, *SD* = 5.62), recruited from the School of Psychology’s participant panel. They were remunerated according to the random lottery incentive system, which has been shown to elicit true preferences and to have other desirable properties (Cubitt, Starmer & Sugden, 1998; Lee, 2008; Starmer & Sugden, 1991). We paid every participant a show-up fee of £5.00 ($8.00) plus an additional amount, up to £5.00 more, corresponding to their payoffs in a randomly pre-selected game. To maximize the incentive value of the remuneration, we did not mention the show-up fee until participants had completed the experiment: before and during the experiment they knew only that they could earn up to £10.00 ($16.00).

Avoiding arbitrary game construction for reasons explained in Experiment 1, we devised 14 symmetric 3 × 3 games, seven S-soluble and seven non-S-soluble, starting with a template containing fixed payoffs in the main diagonal only: (3, 3) in the (*A*, *A*) cell, (2, 2) in the (*B*, *B*) cell, and (1, 1) in the (*C*, *C*) cell. The remaining cells were then populated with payoffs from the set {0,4,5}, avoiding any strongly or weakly dominant strategies and maintaining symmetry in every game. The seven S-soluble and seven non-S-soluble games used in the experiment are displayed in Figs. 5 and 6.

To control for order effects, and to enable comparisons between games with different permutations of rows and columns, we created 10 distinct sets of experimental games from among 36 × 14! ≈ 3.14 × 10^12^ possibilities. First, we permuted the rows and columns of the payoff matrices, a procedure that generates 3! × 3! = 36 permutations for each game. We compiled four sets of materials, each containing a different randomly selected permutation of each of the 14 games, plus a fifth set containing all 14 games in root position. Finally, we arranged the 14 games in each set in a different randomized order, and we created a further five sets by reversing the order, to enable order effects to be investigated.

The experiment was conducted over five one-hour testing sessions. Participants were tested in groups of approximately 20–30, seated at computer monitors. They were presented with the on-screen instructions via the SurveyGizmo online survey software tool. The wording is given in Appendix A.

Participants made one-off strategy choices in each of the 14 games, without feedback, choosing from the options *A*, *B*, or *C*. They indicated their choices by clicking radio buttons and recorded the reasons for their choices by typing in open text boxes below each payoff matrix. They were able to change their strategy choice and reasons for their choice until they hit the submit button to move on to the next game (returning to previous games was not allowed). After the participants had completed all 14 games, data were downloaded from SurveyGizmo into a pre-programmed Microsoft Excel spreadsheet. For calculation of payoffs, players were paired consecutively in the order in which they had logged on to their computers, and their payoffs were then automatically calculated for a randomly preselected game. Participants were thanked and paid what they had earned before they left the laboratory.

### Results^4^ 4Raw data for Experiment 2 are available in the Supplementary Materials.


Following the procedure described in relation to Experiment 1, we performed a binary logistic regression analysis to predict Stackelberg strategy choices (1 = Stackelberg strategy choice, 0 = non-Stackelberg strategy choice), using participant identity, participant age, participant sex, game number, value of the payoff if both players choose Stackelberg strategies, and number of equilibria in the game as predictor variables. After fitting the model, we tested it against a constant model, and the difference was statistically significant: *χ*^2^(16) = 411.14, *p* < .001. Once again, the value of the Nagelkerke *R*^2^ = .277 indicates that the strength of relationship between model predictions and Stackelberg strategy choices is medium. The proportion of correct predictions was 72% overall (67% for choice of non-Stackelberg strategies and 75% for choices of Stackelberg strategies). As in Experiment 1, only game number contributed significantly to the prediction, Wald *χ*^2^(13) = 312.73, *p* < .001, with some games eliciting significantly more Stackelberg strategy choices than others. Replicating the model fitting results in Experiment 1, participant variables (identity, age, and sex) did not show any significant contribution to model predictions independent of game differences, and neither did other game variables (the value of the payoff if both players choose Stackelberg strategies and the number of equilibria in the game).

The main results of Experiment 2 for S-soluble games are shown in Table 3, together with the results of chi-square goodness-of-fit tests against the null hypothesis of one-third Stackelberg strategy choices and two-thirds other strategies, and effect sizes estimated using Cohen’s index *w* (Cohen, 1992). In six of the seven S-soluble games (Games 1, 2, 3, 4, 6, 7), more than one-third of participants chose the options associated with the Stackelberg strategy. In each of these games, the effect is highly significant, and in five of the six, Cohen’s effect size index *w* is large; in the sixth (Game 6), the bias toward the Stackelberg strategy is significant but the effect size is small. In Game 5, there was no significant difference in the frequencies with which the strategies were chosen. In spite of this atypical game, the results provide substantial evidence that players may have been influenced by strong Stackelberg reasoning in S-soluble games.

**Table 3:** Strategy choices in S-soluble games: Experiment 2 (N = 127).

Game	S strategy	% *A*	% *B*	% *C*	*χ*^2^(1)	*p*<	*w*
1	A	74.80	20.47	4.72	100.38	.001	0.89
2	A	88.98	7.09	3.94	179.98	.001	1.90
3	A	93.70	4.72	1.57	211.64	.001	1.29
4	A	68.50	14.96	16.54	72.40	.001	0.76
5	C	37.80	34.65	27.56	1.70	*ns*	0.11
6	A	44.88	47.24	7.87	8.11	.004	0.25
7	A	73.23	23.62	3.15	92.96	.001	0.73

**Notes.**

Cohen’s (1992) effect size index *w* ≥ 0.10 is small, *w* ≥ 0.30 is medium, *w* ≥ 0.50 is large.

Results for the non-S-soluble games, shown in Table 4, reveal variable strategy choices, with a significant bias toward Stackelberg strategies in three games (Games 9, 11, and 13) and a non-significant bias away from Stackelberg strategies in the remaining four (Games 8, 10, 12, and 14). The mean effect size *w* (0.36) is much smaller than in the S-soluble games (0.85). Players chose Stackelberg strategies more frequently in S-soluble games (*M* = 67.38%) than in non-S-soluble games (*M* = 46.26%), replicating a finding from Experiment 1, and in this case the difference is significant: *t*(126) = 8.62, *p* < .001.

**Table 4:** Strategy choices in non-S-soluble games: Experiment 2 (N = 127).

Game	S strategy	% *A*	% *B*	% *C*	*χ*^2^(1)	*p*<	*w*
8	C	14.17	59.84	25.98	2.83	*ns*	0.15
9	A	70.87	26.77	2.36	82.36	.001	0.81
10	B	60.63	36.22	3.15	0.60	*ns*	0.07
11	B	17.32	59.06	23.62	38.99	.001	0.55
12	C	68.25	3.97	27.78	1.55	*ns*	0.11
13	A	70.87	3.15	25.98	82.36	.001	0.81
14	B	54.33	33.07	12.60	0.00	*ns*	0.00

**Notes.**

Cohen’s (1992) effect size index *w* ≥ 0.10 is small, *w* ≥ 0.30 is medium, *w* ≥ 0.50 is large.

A mixed 2 (S-soluble versus non-S-soluble) × 2 (root position versus permuted matrix) ANOVA was carried out, with the dependent variable being the number of times that participants chose Stackelberg strategies. They chose Stackelberg strategies significantly more frequently in the S-soluble games (*M* = 4.72, *SD* = 1.40) than in the non-S-soluble games (*M* = 3.24, *SD* = 1.34), *F*(1, 125) = 73.756, *p* < .001, partial *η*^2^ = .37). There was no significant influence of whether the game was in root position or permuted, *F*(1, 125) = 1.414, *p* = .237, *ns*, and there was no significant interaction (*p* = .946).

To check for possible order effects, we examined the distributions of Stackelberg versus non-Stackelberg strategy choices, game by game. The means did not differ significantly between the first half and the second half of the testing session for any S-soluble or non-S-soluble game. There was thus no evidence for order effects.

A preliminary content analysis of the reasons for strategy choices given by participants revealed eight main categories of reasons. One of the investigators assigned each of the 1,778 reasons (14 for each of 127 participants) to one of the eight categories. In the rare cases in which a participant gave two or more distinct reasons for a choice, only the most strongly emphasized reason was counted, and whenever two or more reasons seemed to be equally emphasized, only the first was counted. A second investigator independently judged and classified 448 (>25%) of the reasons to enable an inter-rater reliability check to be performed. Cohen’s Kappa yielded a value of *K* = .88 (*p* < .001), confirming very high inter-rater reliability.

The eight categories of reasons are as follows (typical examples from the participants’ verbal responses are shown in parentheses).

1. Sequential reasoning or mind reading: choosing a strategy on the basis of a guess or inference about the co-player’s likely choice, and trying to maximize own payoff on that basis (“The red person may choose B for highest return so I choose A”; “They will choose C to maximize their winnings. Because there is a total of 8 to be had, therefore I choose A to maximize mine”)
2. Joint payoff maximization: choosing a strategy that maximizes the total payoff of the pair (“Maximise both our points”; “It’s the highest payoff we both can get”)
3. Choosing randomly (“No reason just random choice”)
4. Simple expected utility maximization: choosing a strategy that maximizes the average or expected payoff if the co-player is equally likely to choose any counterstrategy (“Chance to win 0 is 66.7% in all the options, but A provides an opportunity to win higher score”; “A or C is better than B, but A can get higher scores”)
5. Relative payoff maximization: choosing a strategy with the aim of beating the co-player (“Either I win or we both lose”; “More than red”)
6. Equality-seeking (“Equal amount of points won”; “We get more or less the same amount which is fair”)
7. Maximax: choosing a strategy that provides the possibility of receiving the highest possible payoff in the game (“It has the highest blue number”; “I thought I would try to be greedy and try to pick the highest amount”)
8. Ambiguous or unclassifiable (“It’s more objective”; “If I choose A I will have more than 50% chance to get marks”)

The distributions of reasons for choice are shown in Table 5 for S-soluble games and in Table 6 for non-S-soluble games. The frequencies deviate significantly from chance in all 14 games, with large effect sizes (*w* > .50) in every game. Most frequently cited was Reason 1 (required by strong Stackelberg reasoning) in both S-soluble games (*M* = 24.18%) and non-S-soluble games (*M* = 31.04%), followed by Reason 4 (required by cognitive hierarchy Level-1 reasoning) in both S-soluble games (*M* = 19.57%) and non-S-soluble games (*M* = 20.14%), followed by Reason 6 (equality-seeking) in S-soluble games (*M* = 17.32%) and Reason 2 (required for team reasoning) in non-S-soluble games (*M* = 14.62%). Although Reason 1 is required by strong Stackelberg reasoning, on its own it provides only weak evidence for the theory, because it would be required by other forms of strategic reasoning as well.

**Table 5:** Distribution of reasons for choice across games: S-soluble games, Experiment 2.

	Reasons for choice																		
	1		2		3		4		5		6		7		8				
Game	*N*	**%**	*N*	**%**	*N*	**%**	*N*	**%**	*N*	**%**	*N*	**%**	*N*	**%**	*N*	**%**	*χ*^2^(7)	*p*<	*w*
1	24	18.90	18	14.17	2	1.57	21	16.54	3	2.36	27	21.26	9	7.09	23	18.11	42.64	.001	0.58
2	39	30.71	13	10.24	1	0.79	22	17.32	3	2.36	22	17.32	11	8.66	16	12.60	64.81	.001	0.71
3	25	19.69	43	33.86	2	1.57	15	11.81	0	0.00	17	13.39	14	11.02	11	8.66	55.39	.001	0.66
4	31	24.41	15	11.81	1	0.79	34	26.77	4	3.15	19	14.96	6	4.72	17	13.39	64.81	.001	0.71
5	29	22.83	16	12.60	1	0.79	27	21.26	7	5.51	21	16.54	11	8.66	15	11.81	40.75	.001	0.57
6	31	24.41	14	11.02	1	0.79	31	24.41	2	1.57	22	17.32	10	7.87	16	12.60	59.66	.001	0.68
7	36	28.35	13	10.24	3	2.36	24	18.90	4	3.15	26	20.47	6	4.72	15	11.81	62.17	.001	0.70
Mean		24.18		14.84		1.24		19.57		2.58		17.32		7.53		12.71			

**Table 6:** Distribution of reasons for choice across games: non-S-soluble games, Experiment 2.

	Reasons for choice																		
	1		2		3		4		5		6		7		8				
Game	*N*	**%**	*N*	**%**	*N*	**%**	*N*	**%**	*N*	**%**	*N*	**%**	*N*	**%**	*N*	**%**	*χ* ^2^	*p*<	*w*
8	38	29.92	19	14.96	2	1.57	26	20.47	2	1.57	19	14.96	7	5.51	14	11.02	67.96	.001	0.73
9	41	32.28	12	9.45	1	0.79	29	22.83	4	3.15	12	9.45	11	8.66	17	13.39	76.91	.001	0.78
10	46	36.22	18	14.17	1	0.79	26	20.47	4	3.15	11	8.66	8	6.30	13	10.24	92.65	.001	0.85
11	34	26.77	18	14.17	2	1.57	28	22.05	2	1.57	16	12.60	9	7.09	18	14.17	57.76	.001	0.67
12	35	27.56	18	14.17	3	2.36	25	19.69	4	3.15	16	12.60	8	6.30	18	14.17	52.09	.001	0.64
13	43	33.86	25	19.69	1	0.79	25	19.69	2	1.57	12	9.45	8	6.30	11	8.66	89.25	.001	0.84
14	39	30.71	20	15.75	1	0.79	20	15.75	5	3.94	17	13.39	5	3.94	20	15.75	65.82	.001	0.72
Mean		31.04		14.62		1.24		20.14		2.58		11.59		6.30		12.48			

Tables 7 and 8 show reasons classified by strategy choices in S-soluble and non-S-soluble games respectively. In the S-soluble games, the largest number of players who chose Stackelberg strategies gave Reason 1 as the reason for their choices (26.42%). The next most common reason for choosing Stackelberg strategies was Reason 6 (19.09%), followed by Reason 2 (16.06%) and Reason 4 (15.06%). However, in the simplest game of all, Game 3, which is essentially a 3 × 3 Hi-Lo game, the most common reason for choosing the *A* strategy was Reason 2 (36.13%), followed by Reason 1 (21.01%), Reason 6 (13.45%), and Reason 4 (12.61%).

**Table 7:** Reasons classified by choices made in S-soluble games (percentages), Experiment 2.

		Reasons for choices							
Game	Choice	1	2	3	4	5	6	7	8
1	*A* ^a^	20.00	16.84	0.00	17.89	3.16	23.16	8.42	10.53
	*B*	15.38	7.69	3.85	15.38	0.00	19.23	3.85	34.62
	*C*	16.67	0.00	16.67	0.00	0.00	0.00	0.00	66.67
2	*A* ^a^	30.97	11.50	0.88	18.58	2.65	17.70	9.73	7.96
	*B*	33.33	0.00	0.00	11.11	0.00	0.00	0.00	55.56
	*C*	20.00	0.00	0.00	0.00	0.00	40.00	0.00	40.00
3	*A* ^a^	21.01	36.13	0.84	12.61	0.00	13.45	11.76	4.20
	*B*	0.00	0.00	16.67	0.00	0.00	16.67	0.00	66.67
	*C*	0.00	0.00	0.00	0.00	0.00	0.00	0.00	100.00
4	*A* ^a^	26.44	12.64	1.15	20.69	4.60	18.39	6.90	9.20
	*B*	21.05	15.79	0.00	21.05	0.00	5.26	0.00	36.84
	*C*	19.05	4.76	0.00	57.14	0.00	9.52	0.00	9.52
5	*A*	20.83	25.00	2.08	0.00	0.00	35.42	0.00	16.67
	*B*	13.64	9.09	0.00	52.27	2.27	9.09	0.00	13.64
	*C* ^a^	37.14	0.00	0.00	11.43	17.14	0.00	31.43	2.86
6	*A* ^a^	19.30	24.56	1.75	7.02	0.00	35.09	0.00	12.28
	*B*	33.33	0.00	0.00	41.67	3.33	0.00	16.67	5.00
	*C*	0.00	0.00	0.00	20.00	0.00	20.00	0.00	60.00
7	*A* ^a^	30.11	10.75	2.15	17.20	3.23	25.81	0.00	10.75
	*B*	26.67	10.00	3.33	20.00	3.33	6.67	20.00	10.00
	*C*	0.00	0.00	0.00	50.00	0.00	0.00	0.00	50.00

a Stackelberg strategy

**Table 8:** Reasons classified by choices made in non-S-soluble games (percentages), Experiment 2.

		Reasons for choices							
Game	Choice	1	2	3	4	5	6	7	8
8	*A*	27.78	5.56	0.00	0.00	0.00	55.56	0.00	11.11
	*B*	34.21	19.74	0.00	26.32	0.00	9.21	0.00	10.53
	*C* ^a^	21.21	9.09	6.06	18.18	6.06	6.06	21.21	12.12
9	*A* ^a^	23.33	10.00	1.11	27.78	4.44	12.22	12.22	8.89
	*B*	55.88	8.82	0.00	11.76	0.00	2.94	0.00	20.59
	*C*	33.33	0.00	0.00	0.00	0.00	0.00	0.00	66.67
10	*A*	36.36	15.58	0.00	24.68	0.00	11.69	0.00	11.69
	*B* ^a^	34.78	13.04	2.17	15.22	8.70	2.17	17.39	6.52
	*C*	50.00	0.00	0.00	0.00	0.00	25.00	0.00	25.00
11	*A*	4.55	18.18	0.00	4.55	0.00	50.00	0.00	22.73
	*B* ^a^	20.00	12.00	1.33	32.00	2.67	6.67	12.00	13.33
	*C*	60.00	16.67	3.33	10.00	0.00	0.00	0.00	10.00
12	*A*	31.40	17.44	1.16	23.26	0.00	15.12	1.16	10.47
	*B*	0.00	0.00	20.00	0.00	0.00	20.00	0.00	60.00
	*C* ^a^	22.86	8.57	2.86	14.29	11.43	5.71	20.00	14.29
13	*A* ^a^	27.78	20.00	0.00	27.78	2.22	10.00	8.89	3.33
	*B*	25.00	0.00	0.00	0.00	0.00	50.00	0.00	25.00
	*C*	51.52	21.21	3.03	0.00	0.00	3.03	0.00	21.21
14	*A*	26.09	18.84	1.45	23.19	1.45	14.49	5.80	8.70
	*B* ^a^	38.10	16.67	0.00	4.76	2.38	16.67	0.00	21.43
	*C*	31.25	0.00	0.00	12.50	18.75	0.00	6.25	31.25

a Stackelberg strategy

The results of Experiment 2 corroborate those of Experiment 1 and provide more persuasive evidence for strong Stackelberg reasoning. A significant bias toward Stackelberg strategies occurred in six of the seven S-soluble games, with large effect sizes in all but one of those games. In non-S-soluble games, effect sizes were much smaller, as expected, and choices were biased toward Stackelberg strategies in half these games and away from Stackelberg strategies in the rest. The anomalous S-soluble game was Game 5, in which no significant deviation from chance occurred. Once again, it is easy to explain the anomaly, because Game 5 is the only S-soluble game in which the Stackelberg solution yields very small (1, 1) payoffs. These findings suggest, once again, that players are influenced by strong Stackelberg reasoning but are reluctant to choose Stackelberg strategies when the associated rewards are very small. It is also noteworthy that Game 5 is the only S-soluble game in which the sum of payoffs to the two players is not greater than in any other outcome, suggesting (as in Experiment 1) that collective rationality or team reasoning may have influenced the players’ strategy choices in these games. As noted in relation to the results of Experiment 1, there is evidence from previous research with asymmetric games (Colman, Pulford & Lawrence, *in* *press*) that players use both strong Stackelberg reasoning and team reasoning, but the symmetric games described in this article cannot distinguish between the two methods of reasoning.

The most frequent reason given by the players for their strategy choices, in both S-soluble and non-S-soluble games, was sequential reasoning or mind reading. Given that this is what is required for strong Stackelberg reasoning, these qualitative data provide additional, weakly corroborative evidence that strong Stackelberg reasoning influenced at least some of the players in some of the games.

## Discussion

Although strategic coordination is a ubiquitous feature of social interaction, orthodox game theory cannot explain it satisfactorily. In particular, orthodox game theory cannot justify the choice of strategies associated with payoff-dominant Nash equilibria in common interest games, nor can it explain the powerful intuition that it is rational to choose the component strategies of such equilibria. The theory of strong Stackelberg reasoning offers a potential explanation, and the experiments reported in this article suggest that it is quite powerful in explaining strategy choices in a wide variety of 3 × 3 games. Our results replicate and extend the findings of an earlier experiment using symmetric 2 × 2 games (Colman & Stirk, 1998) and another using asymmetric 3 × 3 and 4 × 4 games (Colman, Pulford & Lawrence, *in* *press*). Given the technical limitations of symmetric 2 × 2 games for testing the theory, especially confounding with strategic dominance, our results provide the first evidence for choices associated with strong Stackelberg reasoning in symmetric games.

The choice data in Experiments 1 and 2, and the qualitative reasons for choice in Experiment 2, taken together, suggest that some players used strong Stackelberg reasoning, or a form of reasoning functionally equivalent to it, in S-soluble games but were much less strongly attracted to it in non-S-soluble games. The findings of both experiments also suggest that players who might otherwise have used strong Stackelberg reasoning tended to abandon it in favor of simple expected utility maximization (equivalent to cognitive hierarchy Level-1 reasoning), equality seeking, or joint payoff maximization (a requirement of team reasoning) when they were unimpressed with the payoffs offered by strong Stackelberg reasoning. In both experiments, players were reluctant to follow through with strong Stackelberg reasoning when it mandated strategy choices associated with very small payoffs, or where the sum of payoffs to both players was greater in another outcome of the game. This is reminiscent of the well-known reluctance of many experimental participants to choose dominant strategies in social dilemmas, in which the sum of payoffs to the players is greater in an outcome achieved by playing non-dominant strategies (Balliet, Mulder & Van Lange, 2011; Colman, 2003).

The theory of strong Stackelberg reasoning assumes that players act as though their co-players could anticipate their strategy choices, or as though the co-players could move second with foreknowledge of their preceding move. If players actually believed that their actions could be anticipated in this way in simultaneous, independent-choice games, then such beliefs would be literally false; but game theory imposes no restrictions on the beliefs and preferences of players, apart from consistency requirements. Furthermore, it is not a requirement of the theory that players actually believe that their choices will be anticipated; the theory is applicable even if they use this assumption merely as a heuristic device in certain strategic situations, knowing full well that their co-players cannot in reality anticipate their choices. This is essentially the form of reasoning used by von Neumann and Morgenstern in their analysis of strictly competitive games (Von Neumann & Morgenstern, 1944). The theory of strong Stackelberg reasoning can therefore be assimilated into existing theory, unlike other explanations, and it also has the advantage over some alternative explanations that it solves all—rather than just some—common interest games.

A valid criticism of our experiments is that the option choices that are consistent with strong Stackelberg reasoning are also consistent with other reasoning processes. Although this is unavoidable in symmetric games, it is worth commenting in particular on the leading alternatives to strong Stackelberg reasoning, namely team reasoning and cognitive hierarchy Level-1 reasoning. In Experiment 1, team reasoning, or Reason 2 among the reasons for choice elicited from participants in Experiment 2 (“choosing a strategy that maximizes the total payoff of the pair”), makes the same unique prediction as strong Stackelberg reasoning in four of the six S-soluble games, and in Experiment 2, it makes the same unique prediction as strong Stackelberg reasoning in five of the seven S-soluble games. In Experiment 1, Cognitive hierarchy Level-1 reasoning, or Reason 4 (“choosing a strategy that maximizes the average or expected payoff if the co-player is equally likely to choose any counterstrategy”), makes the same unique prediction as strong Stackelberg reasoning in three of the six S-soluble games, and in Experiment 2 it makes the same unique prediction as strong Stackelberg reasoning in four of the seven S-soluble games. In spite of these inevitable overlaps, our experiments provide two additional lines of evidence of the distinct influence of strong Stackelberg reasoning. First, players chose Stackelberg strategies significantly more frequently in S-soluble than non-S-soluble games in both experiments—a finding predicted only by the theory of strong Stackelberg reasoning. Second, the most frequent reason for choice elicited in Experiment 2 was sequential reasoning or mind reading, and this too is compatible with strong Stackelberg reasoning but not with team reasoning or cognitive hierarchy Level-1 reasoning. Furthermore, the findings of Colman, Pulford & Lawrence (*in* *press*), in which the use of asymmetric experimental games ensured that different theories predicted different choices, provide independent evidence for frequent use of strong Stackelberg reasoning and also team reasoning and cognitive hierarchy Level-1 reasoning.

Our results corroborate those of other studies of strong Stackelberg reasoning in symmetric 2 × 2 and asymmetric 3 × 3 and 4 × 4 games (Colman, Pulford & Lawrence, *in* *press*; Colman & Stirk, 1998). Evidence has been reported that team reasoning and cognitive hierarchy Level-1 reasoning also influence decision making in coordination games (Bardsley *et al.*, 2010; Colman, Pulford & Lawrence, *in* *press*; Colman, Pulford & Rose, 2008). There is also evidence that players frequently consider two or more of these forms of reasoning before reaching decisions and may have used strong Stackelberg reasoning more frequently in simpler games than in 4 × 4 games, in which the cognitive burden and working memory demands of calculating and checking Stackelberg strategies is greater (Colman, Pulford & Lawrence, *in* *press*). However, it is possible that players may be more inclined to use strong Stackelberg reasoning in complicated games when there is more at stake than the modest financial remuneration of an experimental game.

## Supplemental Information

10.7717/peerj.263/supp-1 Supplemental Information 1 The Dataset from Experiment 1

10.7717/peerj.263/supp-2 Supplemental Information 2 The Dataset from Experiment 2

## Appendix A. Instructions in Experiments 1 and 2

### Experiment 1

Players were presented with the following written instructions:

You will be presented with a series of 12 grids. On each grid you will be asked to make a choice between A, B, or C. You will be choosing between either the rows (if you have been assigned the color red) or the columns (if you have been assigned the color blue). The other member of your pair will be presented with the identical grids and will also choose between A, B, or C, in each case using the other color. Your objective is to maximize the number of points and therefore the amount of money that you gain for yourself. The numbers in your assigned color represent your points. The minimum in each grid is zero and the maximum is 5. Your average gain over the 12 grids will be converted into pounds sterling, so you can end up gaining anything from zero to £5. When you will make your choices, you will not know what the other person has chosen. You may not make any attempt to signal to the other person. You will be told what the other person chose after both of you have made your choices. For each grid, please note its number, record your choice by circling either A, B, or C opposite the corresponding grid number on the answer sheet.

After the participants had read the instructions, a sample matrix, similar to the experimental games, was displayed, and participants were asked a question in the form: “If you chose B and the other person chose C, what would you receive and what would the other person receive?” As it turned out, all of our participants were able to answer the test question correctly. The experimental games were then presented on paper, one by one, in the form of colored payoff matrices accompanied by full verbal summaries, along the lines of the following example (relating to Game 1):

If you are Blue and choose A, then:

If Red chooses A, you will get 3, and Red will get 3

If Red chooses B, you will get 5, and Red will get 0

If Red chooses C, you will get 0, and Red will get 0

If you are Blue and choose B, then:

If Red chooses A, you will get 0, and Red will get 5

... and so on.

The words *Red* and *Blue* were altered where appropriate to match the player’s perspective. Participants recorded their choice, received feedback about their co-player’s choice by being shown a card showing A or B or C, and moved on to the next game.

### Experiment 2

Players were presented with the following on-screen instructions:

You will be presented with a series of 14 grids. For each grid you will be asked to choose between A, B and C. You will be paired with a different experimental participant for each of your 14 decisions. In each case, the other participant will be presented with the identical grid and will also be choosing between A, B and C. Your objective for each grid will be to maximize the number of points that you score. At the end of the experiment, one of the grids will be chosen randomly from the 14. The number of points that you and the other participant scored in that grid will be converted to pounds Sterling, and you will be paid that in cash at the end of today’s session. When you are making your choices, you will not know whom you are paired with or what choices they are making. For each grid, please indicate your choice by selecting either A, B or C, and type a few words in the space provided indicating why you made the choice that you did.

The participants were told that they would be randomly paired with another unidentified participant in the room for each of the 14 games and were given the opportunity to seek clarification of anything they did not understand, after which payoff matrices were presented one by one on their monitors, with Player I’s labels and payoffs shown in blue and Player II’s in red. Capitalizing on the fact that the experimental games were symmetric, we assigned all participants to the role of Player I and interpreted their co-players’ choices as though they were in the role of Player II. Because the games are symmetric, this makes no difference to strategy choices and outcomes. The following text was displayed below each payoff matrix to help the participant to interpret the game: “You are the Blue decision maker, choosing between the rows marked *A*, *B*, or *C*. The person you have been paired with is the Red decision maker, choosing between columns *A*, *B*, or *C*. Depending on what you and the other decision maker choose, you will get one of the blue payoffs, and the red decision maker will get one of the red payoffs.” This was followed by a full textual summary of the information shown in the payoff matrix, as in Experiment 1.

## Appendix B. Regression Tables for Experiments 1 and 2

See Tables 9 and 10.

**Table 9:** Regression Table for Experiment 1.

Variables in the Equation						
	*B*	*SE*	Wald	*df*	*p*<	Exp (*B*)
ID	−.007	.004	2.931	1	.087	.993
Age	.025	.014	3.262	1	.071	1.025
Sex (1)	.277	.191	2.108	1	.147	1.320
Game			120.230	9	.001	
Game (1)	1.583	.449	12.438	1	.001	4.871
Game (2)	−1.871	.381	24.065	1	.001	.154
Game (3)	1.327	.421	9.945	1	.002	3.769
Game (4)	−.977	.346	7.988	1	.005	.377
Game (5)	.751	.376	3.989	1	.046	2.118
Game (6)	.834	.381	4.789	1	.029	2.301
Game (4)	−.631	.342	3.413	1	.065	.532
Game (8)	.671	.371	3.267	1	.071	1.956
Game (9)	−.462	.341	1.830	1	.176	.630
Constant	.156	.395	.156	1	.693	1.169

**Notes.**

Variable(s) entered on step 1: ID, Age, Sex, Game.

**Table 10:** Regression Table for Experiment 2.

Variables in the equation						
	*B*	*SE*	Wald	*df*	*p*<	Exp (*B*)
ID	−.002	.002	1.062	1	.303	.998
Age	.005	.010	.221	1	.638	1.005
Sex (1)	−.132	.126	1.087	1	.297	.876
Game			312.733	13	.001	
Game (1)	1.796	.278	41.643	1	.001	6.027
Game (2)	2.798	.341	67.470	1	.001	16.405
Game (3)	3.409	.411	68.724	1	.001	30.245
Game (4)	1.485	.269	30.532	1	.001	4.414
Game (5)	−.262	.274	.913	1	.339	.770
Game (6)	.500	.260	3.709	1	.054	1.649
Game (4)	1.714	.275	38.733	1	.001	5.553
Game (8)	−.342	.277	1.529	1	.216	.710
Game (9)	1.597	.272	34.527	1	.001	4.937
Game (10)	.139	.264	.279	1	.598	1.150
Game (11)	1.073	.261	16.872	1	.001	2.925
Game (12)	−.250	.274	.830	1	.362	.779
Game (13)	1.597	.272	34.527	1	.001	4.937
Constant	−.678	.299	5.161	1	.023	.507

**Notes.**

Variable(s) entered on step 1: ID, Age, Sex, Game.
